# Evidence of a metabolic memory to early-life dietary restriction in male C57BL/6 mice

**DOI:** 10.1186/2046-2395-1-2

**Published:** 2012-09-03

**Authors:** Colin Selman, Sarah Hempenstall

**Affiliations:** 1 Integrative and Environmental Physiology, Institute of Biological and Environmental Sciences, University of Aberdeen, Aberdeen,, AB24 2TZ, UK

**Keywords:** Dietary restriction, Glucose tolerance, Insulin sensitivity, Dietary switch, C57BL/6 mice, Body composition, Insulin, IGF-1, Metabolic memory, Glycemia

## Abstract

**Background:**

Dietary restriction (DR) extends lifespan and induces beneficial metabolic effects in many animals. What is far less clear is whether animals retain a metabolic memory to previous DR exposure, that is, can early-life DR preserve beneficial metabolic effects later in life even after the resumption of *ad libitum* (AL) feeding. We examined a range of metabolic parameters (body mass, body composition (lean and fat mass), glucose tolerance, fed blood glucose, fasting plasma insulin and insulin-like growth factor 1 (IGF-1), insulin sensitivity) in male C57BL/6 mice dietary switched from DR to AL (DR-AL) at 11 months of age (mid life). The converse switch (AL-DR) was also undertaken at this time. We then compared metabolic parameters of the switched mice to one another and to age-matched mice maintained exclusively on an AL or DR diet from early life (3 months of age) at 1 month, 6 months or 10 months post switch.

**Results:**

Male mice dietary switched from AL-DR in mid life adopted the metabolic phenotype of mice exposed to DR from early life, so by the 10-month timepoint the AL-DR mice overlapped significantly with the DR mice in terms of their metabolic phenotype. Those animals switched from DR-AL in mid life showed clear evidence of a glycemic memory, with significantly improved glucose tolerance relative to mice maintained exclusively on AL feeding from early life. This difference in glucose tolerance was still apparent 10 months after the dietary switch, despite body mass, fasting insulin levels and insulin sensitivity all being similar to AL mice at this time.

**Conclusions:**

Male C57BL/6 mice retain a long-term glycemic memory of early-life DR, in that glucose tolerance is enhanced in mice switched from DR-AL in mid life, relative to AL mice, even 10 months following the dietary switch. These data therefore indicate that the phenotypic benefits of DR are not completely dissipated following a return to AL feeding. The challenge now is to understand the molecular mechanisms underlying these effects, the time course of these effects and whether similar interventions can confer comparable benefits in humans.

## Background

Dietary restriction (DR), a reduction in food intake without malnutrition, is a well established experimental paradigm that extends mean and maximum lifespan in many animals [1]. In addition, chronic DR induces a range of physiological benefits, including enhanced glucose tolerance and increased insulin sensitivity in rodents [2,3], non-human primates [4,5] and humans [6,7]. However, while the positive benefits of DR on healthy lifespan are clear, it is unlikely that lifelong DR is a realistic or achievable intervention for most human beings (for discussion, see [8,9]). What is perhaps more achievable are acute DR interventions, particularly if these short-term periods of DR can successfully elicit favorable metabolic effects and particularly if those benefits are retained even following a subsequent return to *ad libitum* (AL) feeding.

It is well established that acute DR improves a range of metabolic parameters in both rodents [10-12] and humans [13,14]. In mice, acute DR introduced either early (4 or 14 weeks of age, respectively) [15,16] or late in life (19 or 29 months of age, respectively) [17,18] rapidly alters (within weeks) transcriptional profiles to those of mice exposed to long-term DR. In addition, the converse switch in older mice from DR back to AL feeding almost completely reverses the DR-induced gene expression profile back to the AL control profile within 8 weeks [17,18]. Initiating DR in mid to late life generally extends lifespan in rodents (for review see [1], and see also [19]) and delays the onset of tumor incidence [17,20]. However, the lifespan extension effect appears to become less pronounced the later in life that DR is initiated. Adult-onset DR also decreased oxidative stress in rodents [21] and improved glucose tolerance in rats [22]. Similarly, dietary switch experiments (from AL to DR or from DR to AL) in mice indicate that the beneficial effects of DR on certain markers of oxidative stress can be improved (or reversed, respectively) within weeks following the switch [23,24]. Dietary shifts in adulthood from DR to AL (or from AL to DR) in *Drosophila*[25,26] and rats [27], and dietary shifts from AL to DR in adult mice [17] rapidly shifts the mortality risk to the group that they are subsequently switched to. In adult flies, at least, this reversal in mortality risk through dietary switching can occur at any age [26]. It is currently unclear in mice whether a shift from DR to control feeding also shifts the rate of ageing accordingly, although this has been demonstrated clearly to be the case in rats [27].

Interestingly, dietary switching in mid life from DR to AL (or the converse AL to DR) in combination with α-lipoic acid supplementation induced a metabolic memory effect in male rats, in that dietary switched animals additionally supplemented with α-lipoic acid retained the survival trajectory of the initial feeding regime [27]. In contrast, rats shifted from 40% DR (initiated at 6 weeks of age) to AL at 6 months of age had a greater median and maximum (tenth percentile) lifespan compared to AL rats [28], although no demonstrable effect on pathology, including cardiomyopathy, nephropathy and various cancers, was reported relative to AL animals [29]. However, the reverse shift from AL to DR at 6 months of age was as effective as lifelong DR in attenuating the incidence of these pathologies [29]. It is currently unclear whether early-life exposure to DR can induce a lasting beneficial metabolic effect in mice subsequently switched back to AL feeding, that is, do animals retain a ‘metabolic memory’ to previous DR exposure? However, it has been reported that rats shifted from 40% DR to AL at 6 months of age remained significantly lighter than AL rats until they reached approximately 700 days of age [28].

**Figure 1:**
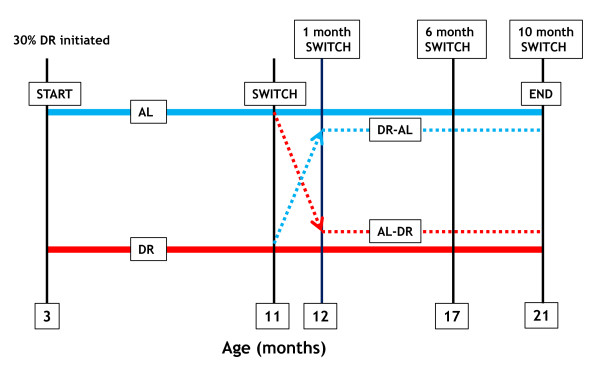
**Schematic showing the experimental switch design.** All mice (male C57BL/6) were fed *ad libitum* (AL) until 2.5 months of age and then dietary restriction (DR) mice underwent a step-down DR regime resulting in 30% DR from 3 months of age onwards. The dietary switch was initiated at 11 months of age, at which time the DR group had been on 30% DR for 8 months. The stippled lines denote the switch groups; either AL to 30% DR (AL-DR) or 30% DR to AL (DR-AL). All metabolic measurements were determined at 1 month, 6 months and 10 months post switch (equivalent to 12 months, 17 months and 21 months of age), except for fasting plasma insulin, fasting plasma insulin-like growth factor 1 (IGF-1) and insulin sensitivity, which were determined at 1 month and 10 months post switch only.

In the present study we examined whether a mid-life dietary switch from AL to DR (AL-DR) or from DR to AL (DR-AL) at 11 months of age (8 months of AL feeding or 30% DR respectively) had any impact on a range of metabolic parameters (for example, body mass, lean/fat mass, glucose tolerance, insulin sensitivity) in male C57BL/6 mice. Figure 1 shows a schematic of the experimental design. Those mice switched from DR-AL at 11 months of age retained significantly enhanced glucose tolerance relative to mice maintained exclusively on an AL diet. This improved glucose tolerance was retained even 10 months following the switch. We also show that mice switched from AL-DR at 11 months of age rapidly adopt the glycemic profile of mice maintained on 30% DR from early life (3 months of age). These data suggest that mice switched from DR-AL in mid life retain a long-term glycemic memory to previous DR exposure, which maintains a long-term improvement in glucose tolerance (and reduction in fasting insulin-like growth factor 1 (IGF-1) levels) relative to mice maintained on an AL diet throughout their life.

## Results

### Body mass, food intake, body composition

**Figure 2:**
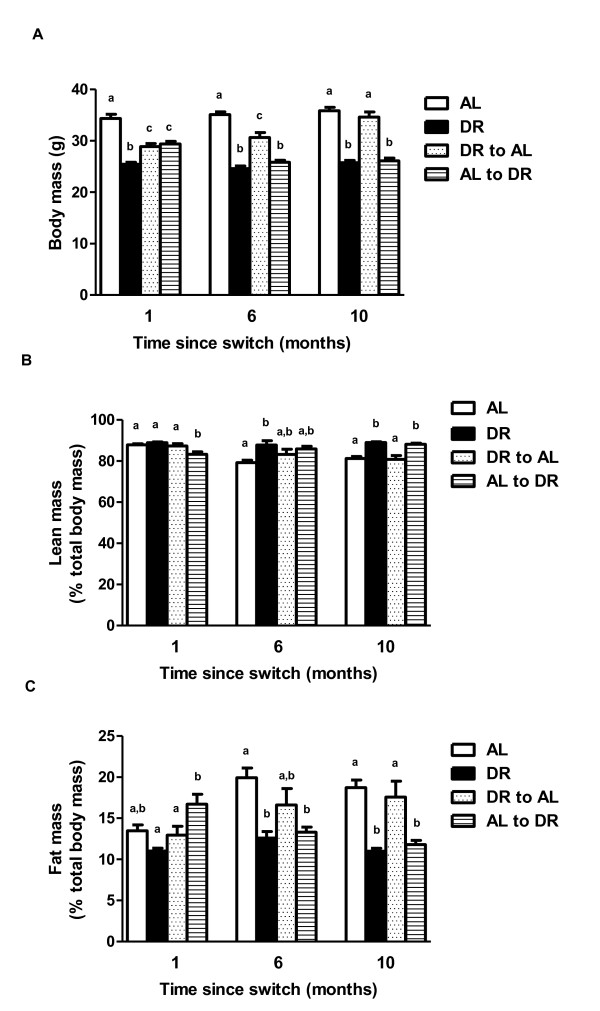
**Body mass and body compositional changes following dietary switches in male C57BL/6 mice.** Body mass **(A)**, percentage lean mass **(B)** and percentage fat mass **(C)** in *ad libitum* (AL), dietary restriction (DR), AL-DR and DR-AL mice at 1 month, 6 months and 10 months post switch. Groups sharing the same letter within the same timepoint (that is, 1 month, 6 months or 10 months) were not significantly different to one another. Results are reported as mean ± standard error of the mean (SEM), with *P* <0.05 regarded as statistically significant. N = 7 to 10 per group.

A significant difference in body mass (BM) was observed between the experimental groups (AL, DR, DR-AL, AL-DR) at 1 month (F = 39.224, *P* <0.001), 6 months (F = 52.744, *P* <0.001) and 10 months (F = 53.715, *P* <0.001) following the dietary switch (Figure 2A). By 1 month after the switch, both the DR-AL and AL-DR groups were significantly lighter than the AL mice, although both switch groups were significantly heavier than the DR mice. At 6 months post switch, the DR-AL switched mice were still significantly lighter than the AL group but significantly heavier than both the DR and AL-DR group. At this same time, the BM of the AL-DR group had decreased to a level similar to the DR group. Only by the 10-month timepoint did the DR-AL mice attain a similar BM to the AL controls, albeit they were still slightly lighter. Perhaps surprisingly, given the time taken for the DR-AL mice to reach a similar BM to the AL mice, these mice had a significantly greater food intake relative to the AL mice at both the 1-month and 6-month timepoints ( Additional file 1). Only by the final timepoint, at which BM was similar, was food intake comparable between these two groups.

The lean mass (as a percentage of total body mass) differed significantly between groups at 1 month (F = 7.890, *P* <0.001), 6 months (F = 3.421, *P* = 0.028) and 10 months (F = 13.346, *P* <0.001) following the switch (Figure 2B). At the 1-month timepoint, the AL-DR mice had a significantly lower lean mass compared with all other groups, with the DR-AL, AL and DR mice all being similar to one another. By 6 months post switch, the DR-AL and AL-DR mice had intermediate, but not significantly different, lean masses to the AL and DR mice. At this same time, the DR group had a significantly greater lean mass compared to AL mice. By the 10-month timepoint, the DR-AL mice had a lean mass indistinguishable from AL mice, with both DR-AL and AL mice having a significantly lower lean mass relative to the AL-DR and DR groups. Percentage fat mass also showed significant groups effects at 1 month (F = 6.942, *P* = 0.001), 6 months (F = 6.228, *P* = 0.002) and 10 months (F = 10.178, *P* <0.001) post switch. After 1 month of the switch, the DR-AL mice had a similar percentage fat mass to the AL and DR mice. However, at this time the fat mass of the AL-DR group was significantly greater than both the DR-AL mice and DR mice, suggesting that there was a preferential loss of lean mass rather than fat mass following initiation of mid-life DR. By 6 months post switch, the DR-AL group had an intermediate fat mass relative to all other groups, with the AL mice having a significantly higher fat mass compared to the DR and AL-DR mice. Following 10 months post switch, the DR-AL group had a similar fat mass to the AL mice, although both of these groups were significantly fatter than either the DR or the AL-DR mice.

### Glucose homeostasis

**Figure 3:**
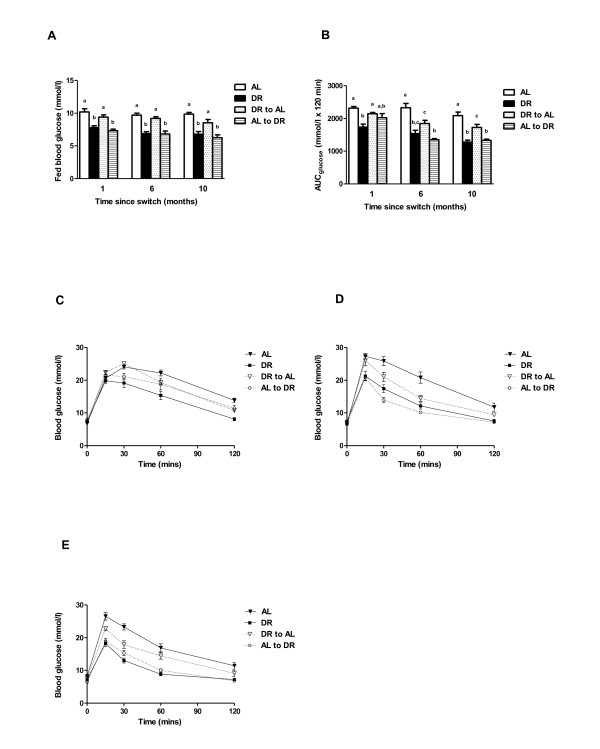
**Fed blood glucose levels and glucose tolerance following dietary switches in male C57BL/6 mice.** Fed blood glucose levels **(A)** and glucose tolerance **(B)**, determined by area under the curve (AUC), in *ad libitum* (AL), dietary restriction (DR), AL-DR and DR-AL mice at 1 month, 6 months and 10 months post switch. Groups sharing the same letter within the same timepoint (that is, 1 month, 6 months or 10 months) were not significantly different to one another. Results are reported as mean ± standard error of the mean (SEM), with *P* <0.05 regarded as statistically significant. In addition, glucose tolerance curves **(C-E)** are shown for all groups at 1 month, 6 months or 10 months post switch, respectively. N = 7 to 10 per group.

In terms of fed blood glucose (Figure 3A), the dietary switch groups rapidly adopted the profiles of the groups they were switched to in mid life, that is, DR-AL and AL were comparable to one another but significantly different to the comparable DR and AL-DR mice within 1 month of the switch (1 month: F = 13.134, *P* <0.001; 6 months: F = 21.298, *P* <0.001; 10 months: F = 15.890, *P* <0.001). Significant group effects on glucose tolerance were also observed at 1 month (F = 10.313, *P* <0.001), 6 months (F = 18.739, *P* <0.001) and 10 months (F = 20.746, *P* <0.001) following the dietary switch (Figure 3B). At 1 month post switch, the DR-AL group had a similar glucose tolerance to AL mice. At this same time, the AL-DR mice had an intermediate glucose tolerance compared to all other groups, with the early-life DR mice having a significantly greater glucose tolerance relative to the DR-AL and AL mice. At 6 months post switch, the DR-AL mice had a significantly enhanced glucose tolerance compared to AL mice. In addition, the DR-AL mice had a glucose tolerance that was not significantly different to the early-life DR group. At this same time the AL-DR mice had a significantly better glucose tolerance compared to both the AL and DR-AL mice. By the 10-month timepoint, the DR-AL mice retained their significantly enhanced glucose tolerance compared to AL mice, although improved glucose tolerance was observed in the DR and AL-DR mice compared to both AL and DR-AL mice at this time. For comparative purposes, the glucose tolerance curves are also shown in Figure 3C-E at the 1-month, 6-month and 10-month timepoints, respectively. This data clearly indicates the significantly improved glucose tolerance of the DR-AL group relative to the AL group at the 6-month and 10-month timepoints. Fasting blood glucose levels were not significantly different between any groups at either the 1-month (F = 1.586, *P* = 0.210) or 6-month timepoints (F = 1.943, *P* = 0.141). However, by 10 months a significant difference between groups was detected (F = 3.639, *P* = 0.024), with the fasting blood glucose of the AL mice being significantly elevated (*P* = 0.021, *post hoc* Tukey) relative to the AL-DR mice.

### Fasting plasma insulin levels, fasting plasma IGF-1 levels and insulin sensitivity

**Figure 4:**
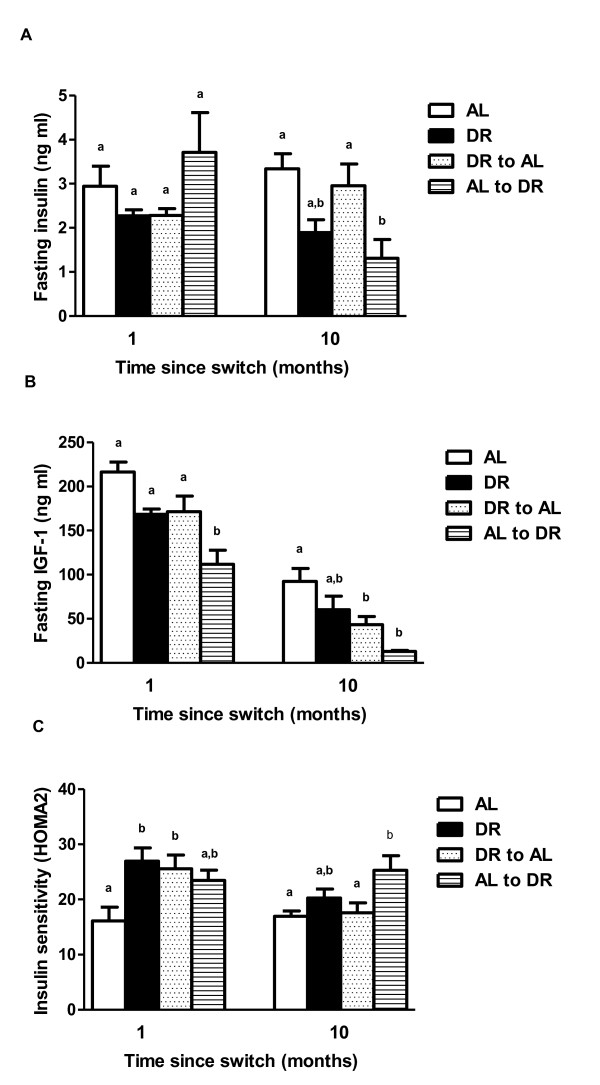
**Fasting plasma insulin, fasting plasma insulin-like growth factor 1 (IGF-1) and insulin sensitivity following dietary switches in male C57BL/6 mice.** Fasting plasma insulin levels **(A)**, fasting plasma IGF-1 levels **(B)** and insulin sensitivity **(C)**, determined using the updated homeostatic model assessment (HOMA2) model in *ad libitum* (AL), dietary restriction (DR), AL-DR and DR-AL mice at 1 month, 6 months or 10 months post switch. Groups sharing the same letter within the same timepoint (that is, 1 month, 6 months or 10 months) were not significantly different to one another. Results are reported as mean ± standard error of the mean (SEM), with *P* <0.05 regarded as statistically significant. N = 7 to 10 per group.

The effects of dietary switching on fasting plasma insulin, fasting plasma IGF-1 and insulin sensitivity (HOMA2) were determine at 1 month and 10 months post switch. Despite fasting plasma insulin levels being lower in DR and DR-AL mice 1 month following the dietary switch compared to AL and AL-DR mice (Figure 4A), no significant group effect was detected (F = 0.971; *P* = 0.426). However, by the 10-month timepoint a significant group effect was seen (F = 5.888; *P* = 0.004), with the DR-AL and AL mice having significantly elevated fasting insulin levels relative to the AL-DR group. The early-life DR group also had lower fasting insulin levels compared to both the DR-AL and AL mice at this time, although this did not quite reach statistical significance when using *post hoc* Tukey tests. A significant group effect on fasting plasma IGF-1 levels was observed at both 1 month (F = 9.274, *P* = 0.001) and 10 months (F = 6.764, *P* = 0.002) post switch (Figure 4B). At the 1-month timepoint, no differences were detected between the DR-AL, AL and DR mice, although the AL-DR group had significantly lower fasting IGF-1 levels compared to all other groups. By 10 months following the switch, both switch groups had significantly lower IGF-1 levels relative to AL mice, with the early-life DR group having an intermediate IGF-1 level relative to all other groups. Finally, insulin sensitivity (Figure 4C) at 1 month post switch was significantly increased in the DR-AL and early-life DR mice relative to AL mice (F = 4.602, *P* = 0.013). Insulin sensitivity in the AL-DR mice at this time was intermediate to all other groups. However, by the 10-month timepoint, the DR-AL mice had comparable insulin sensitivity to the AL group. The AL-DR mice at this time had significantly greater insulin sensitivity than both the AL and DR-AL mice (F = 4.281, *P* = 0.016). Perhaps surprisingly, the early-life DR group had an intermediate insulin sensitivity profile relative to all other groups at this time.

## Discussion

This study examined the effects of a mid life (11 months of age) dietary switch from 30% DR to AL (DR-AL) feeding or from AL feeding to 30% DR (AL-DR) on a range of metabolic parameters and compared these to animals maintained exclusively on AL feeding or 30% DR from early life (3 months of age). In particular we were interested in whether the experience of early-life DR could confer metabolic benefits in mice subsequently switched back to an AL diet, that is, does a metabolic memory exist? Currently, there is very little experimental evidence to suggest that mice retain a metabolic memory to previous DR exposure. A dietary shift from AL-DR in adult flies [25,26], rats [27] and mice [17] resulted in animals rapidly adopting the decreased mortality risk of long-term DR animals. The converse shift in flies [25,26] and rats [27] has also been shown to increase the mortality risk to that of AL animals. In addition, the shift from DR-AL rapidly reverses the majority of the transcriptional changes induced by long-term DR [17,18]. In contrast to the studies above [25-27], a dietary shift from DR-AL in rats demonstrated a benefit to early-life DR on lifespan [28], that is, the DR-AL rats lived significantly longer than animals maintained on AL feeding throughout their life. Similar evidence of a metabolic memory to previous DR exposure was also reported in a second rat study [27]. Interestingly, this study showed that those animals switched from DR-AL (or AL-DR) at either 6 months or 12 months only retained the survival trajectory of the initial feeding regime when they were additionally dietary supplemented with α-lipoic acid [27].

In terms of BM, perhaps surprisingly the mice in the current study shifted from DR and back to AL feeding (DR-AL) remained lighter that AL mice at 1 month and 6 months post switch, only reaching a similar BM to AL mice 10 months after the dietary switch. In addition, those animals switched to DR in mid life (AL-DR), were still heavier than the early-life DR group following 1 month post switch, although by the 6-month timepoint their BM was indistinguishable from the DR group. While it has been reported that a shift back to AL feeding from acute DR (≤ 100 day duration) can increase BM back to AL levels within 50 days in MF1 mice [30], we observed a far slower response in agreement with studies in rats [28] and in other strains of mice [19]. While the shift from DR-AL feeding induces hyperphagia, this response is suggested as being highly transient (1 to 5 days), after which food intake (FI) levels (and BM) return to AL levels [30,31]. However, in rats the mass-adjusted FI of animals switched from DR-AL was elevated until the switch animals reached the similar BM of AL rats, around 18 months post switch [28]. In our study, food intake in the DR-AL group was significantly greater at the 1-month and 6-month timepoints relative to AL mice. This hyperphagic response was not observed in the DR-AL group at the final timepoint, at a time when no BM differences were observed between the DR-AL and AL mice. Our data, as does the rat data [28], suggests that hyperphagia following DR is maintained until BM reaches the levels of AL animals. A similar prolonged hyperphagic response was also reported in male C57BL/6 mice following a dietary shift from DR-AL, although interestingly this was not observed in female mice [32]. Significant differences in body composition (percentage lean and fat mass) were observed between groups throughout the timecourse of this experiment. At the 1-month timepoint, percentage lean mass was lowest, but percentage fat mass highest, in the AL-DR mice. This suggests that this group preferentially maintained their fat mass relative to lean mass following the mid-life switch to DR. The reasons for this are unclear, although may be a temporal stress response to an unpredictable food resource, as previously suggested [33]. Interestingly, this response after 1 month of 30% DR may be age specific, as 3 weeks of 30% DR had no effect on either lean or fat mass in young (15 week old) male C57BL/6 (and DBA/2) mice [10], although it should be noted that this paper used a different method to control for BM differences between groups than used in the current manuscript. It was only after 10 months post switch that the DR-AL and AL-DR groups mirrored the body composition profiles of the early-life AL and DR groups, respectively.

In agreement with previous studies examining short-term DR in young mice [10-12], and in contrast to the body mass and body composition effects observed, mice switched from AL-DR rapidly adopted the improved glucose tolerance of the DR group. This similarity in glucose tolerance to mice exposed to DR from early life, and significant improvement relative to the AL mice, was maintained at both 6 months and 10 months post switch. These data indicate that DR, even when initiated in mid life, can induce significant, and rapid, benefits to the glycemic profile of mice, and support previous data in both rodents [22] and humans [13,14,34]. In mice switched from DR-AL, glucose tolerance was not statistically different to AL mice at 1 month post switch. However, by 6 months and 10 months post switch this group had a significantly enhanced glucose tolerance relative to the AL mice, despite having very similar body mass, body composition and fasting insulin levels to AL mice at the 10-month timepoint. These data suggest that male C57BL/6 mice retain a glycemic memory to previous DR feeding that maintains a long-term benefit to glucose tolerance that is independent of body composition and fasting insulin levels. In support, Cameron *et al*.[32] also reported that male C57BL/6 mice switched from AL to DR (12 months of age) and then back to AL (17 months of age) had improved glucose tolerance relative to lifelong AL mice 5 months after switching back to AL feeding. The effect of dietary switching on fasting insulin levels and insulin sensitivity were less clear in our study, although the AL-DR had significantly lower fasting insulin levels and increased insulin sensitivity relative to both the DR-AL and AL mice 10 months post switch. Improved glucose tolerance following late-life initiation in DR has previously been reported in rats [22], although significant differences in serum insulin levels were only seen following glucose challenge [22]. Fasting IGF-1 levels, which we have previously shown respond rapidly to early-life DR in mice [10], were also decreased significantly following mid-life DR, with the AL-DR group having lower IGF-1 levels compared to all other groups. This suggests that dietary switches to DR both in early life [10] and in mid life can elicit similar rapid changes in IGF-1 levels in mice, and that IGF-1 appears more responsive than insulin to DR [10]. After 10 months post switch, while fasting insulin and IGF-1 levels were reduced and insulin sensitivity increased in the AL-DR group relative to AL mice, only IGF-1 levels were reduced in the DR-AL group compared to the AL group. The exact reasons for this uncoupling in the response of insulin and IGF-1 to mid-life dietary shifts back to AL feeding are currently unclear. We suggest that the precise role of insulin and IGF-1 signaling following mid-life dietary switches now requires further investigation, as late-onset DR in rats improved glucose tolerance without having any impact on insulin signaling within skeletal muscle [22]. In addition, while many studies have examined the transcriptional response to DR in many tissues (see for example [15,16,18,35,36]), information on exactly how DR impacts on pancreatic and β-cell function is currently lacking. We suggest that with our data demonstrating clear evidence of a glycemic memory to previous DR exposure in mice, it is now critical to examine the molecular responses in the pancreas to DR and to mid-life dietary switches.

## Conclusions

In the present study we demonstrate that mid-life DR (initiated from 11 months of age) rapidly improves several metabolic parameters to those of mice exposed to DR from early life. In addition, we also provide evidence of the existence of a glycemic memory to previous DR exposure that confers later-life benefits to mice in terms of improved glucose tolerance. Surprisingly, this effect was not associated with alterations in fasted plasma insulin levels or insulin sensitivity (as determined by HOMA2); although it was associated with significantly lower fasting plasma IGF-1 levels. While the idea of a metabolic memory following DR has previously been voiced [37] following studies in rats [27,28], our study, and that of Cameron *et al*. [32], give the strongest support yet that a metabolic memory exists in mice. These findings suggest that the benefits to previous DR are maintained at the level of the phenotype, despite apparent rapid reversals in transcription following similar dietary switches in mice [17,18,38]. Similar directional dietary shifts in rats extend lifespan relative to AL controls [28], and so enhanced glucose tolerance underlying this lifespan extension is certainly plausible. The challenge now is to identify the biochemical and signaling processes that underlie this improved glucose tolerance, including how early-life DR may impacts on processes such as DNA methylation and post-translational modifications to histones and proteins [39-41]. It is unequivocal that impaired glucose tolerance is associated with a range of age-associated pathologies in humans [42-44]. Therefore, understanding how early-life DR improves later-life glucose tolerance may give useful insights in to understanding, and ultimately intervening, in associated age-related pathologies.

## Methods

### Mice

Inbred male C57BL/6 mice were purchased from a commercial breeder (Charles River Laboratories, Margate, UK) at 1 month of age. Mice were maintained in pairs from 2 months of age onwards in polycarbonate shoebox cages (48 × 15 × 13 cm), with *ad libitum* access to water and standard chow (D12450B, Research Diets Inc., New Brunswick, NJ, USA; protein 20 kcal%, carbohydrate 70 kcal%, fat 10 kcal%). Mice were maintained on a 12 h light/12 h dark cycle (lights on 7.00 am to 7.00 pm) at a housing temperature of 22 ± 2 °C. At 2.5 months of age, weight-matched pairs were assigned to the AL or dietary restricted (DR) groups, with no difference in body mass observed between groups at this time (AL = 25.1 ± 0.5 g, DR = 25.3 ± 0.5 g; F = 3.354, *P* = 0.137). The DR protocol used in this study followed exactly a previously described protocol [10,15]. In brief, daily food intake of the DR mice was reduced to 90% of the AL intake at 2.5 months of age, reduced to 80% of AL intake at 2.75 months of age, and then maintained at 70% of AL food intake from 3 months of age onwards, that is, 30% DR relative to AL controls. Food intake of AL mice was measured weekly (± 0.01 g) and 30% DR calculated from the AL mice intake over the preceding week. DR and AL-DR mice were fed daily between 4.30 pm to 5.30 pm. At 11 months of age, mice randomly selected from the 30% group were dietary switched to AL feeding (that is, DR-AL) and mice from the AL group were switched to 30% DR (that is, AL-DR). See Figure 1 for a schematic of the experimental design, indicating the resulting four groups (AL, DR, DR-AL, AL-DR) following the switch and the timepoints at which the metabolic measurements were collected (1 month, 6 months and 10 months post switch). A significant difference in body mass was observed between the AL and DR mice immediately before the switch (F = 94.526, *P* <0.001). However, using *post hoc* Tukey tests we show that no difference in body mass was observed at this time between the AL mice and those mice subsequently switched AL-DR (34.3 ± 0.8 g and 34.5 ± 0.6 g, respectively), or between DR mice and those animals subsequently switched from DR-AL mice (25.5 ± 0.4 g and 25.1 ± 0.3 g, respectively). Note that the mice dietary switched at 11 months of age were switched immediately to full AL feeding or 30% DR, rather than undergoing any incremental step-down (or step-up) in food intake.

### Body mass, food intake and body composition

Body mass and food intake for all groups was measured at 1 month, 6 months and 10 months post switch using an analytical balance (± 0.01 g). Body composition (lean and fat mass) was also determined at 1 month, 6 months and 10 months post switch using dual-energy X-ray absorptiometry (DXA; Lunar PIXImus mouse densitometer, GE Medical Systems, Slough, UK). In brief, mice were weighed and subsequently anaesthetized using 3.5% isoflurane (Henry Schein Animal Health, Dumfries, UK) by inhalation for the duration of the scanning period (approximately 3.5 per min). Lunar PIXImus 2.10 software was used to calculate percentage lean mass and percentage fat mass in the region of interest (defined as the subcranial body, as recommended by the manufacturer) using a previously described protocol [45].

### Glucose homeostasis

Fed blood glucose was determined in AL and DR-AL mice at 11.00 am and in DR and AL-DR mice at 6.30 pm, as described elsewhere [10]. Glucose tolerance was determined at 1 month, 6 months and 10 months post switch following an overnight fast (7.00 pm to 8.00 am), using a previously described protocol [10]. For both fed blood glucose and glucose tolerance tests, DR mice were fed at approximately 3.00 pm on the day immediately prior to testing [10]. In brief, mice were weighed, injected intraperitoneally with 20% d-glucose (2 g/kg) and then blood glucose levels were collected from tail vein samples and read on a glucometer (OneTouch Ultra, Lifescan, High Wycombe, UK) as previously described [46]. Glucose tolerance is expressed as the area under the curve over a 120-min period following the intraperitoneal injection of d-glucose.

### Fasting plasma insulin levels, IGF-1 levels and insulin sensitivity

At 1 month and 10 months post switch, mice from all four groups were fasted overnight, and then weighed and killed by cervical dislocation. Venous trunk blood was collected and the resultant plasma stored at −80 °C. Fasting plasma insulin levels were measured using a mouse insulin ELISA kit (Crystal Chem Inc., Downers Grove, IL, USA). Insulin sensitivity was estimated using the updated homeostatic model assessment (HOMA2) model [47]. In brief, this model generates approximate values of insulin sensitivity derived from fasting glucose and fasting insulin levels. Although this model has not been validated formally in mice, it has been used widely in experimental studies to determine insulin sensitivity and insulin resistance (see for example [10,48-50]. Fasting plasma IGF-1 levels were determined using a mouse IGF-1 ELISA kit (R&D Systems Europe Ltd., Abingdon, UK). All experiments were carried out following local ethical review (University of Aberdeen, UK), under a license from the UK Home Office and followed the ‘principles of laboratory animal care’ (NIH Publication No. 86–23, revised 1985).

### Statistical analysis

All analyses were performed using SPSS v.18 (SPSS Inc., Chicago, IL, USA) and GraphPad Prism v.5 (GraphPad Inc., La Jolla, CA, USA) software. Data were checked for normality using the Shapiro-Wilks test. Data was analyzed using one-way analysis of variance (ANOVA) with *post hoc* Tukey tests to determine differences between groups within a timepoint. Results are reported as mean ± standard error of the mean (SEM), with *P* <0.05 regarded as statistically significant in all cases. Groups sharing the same letter within a timepoint (that is, 1 month, 6 months or 10 months) were not significantly different to one another. Sample size was 7 to 10 per group throughout.

## Competing interests

The authors declare they have no competing interests.

## Authors’ contributions

SH participated in the design of the study, undertook the experiments described and commented on the manuscript. CS conceived the study, participated in its design and coordination, performed the statistical analysis and wrote the manuscript. Both authors read and approved the final manuscript.

## Supplementary Material

Additional file 1: **Title: Mean (± SEM) daily food intake per cage at 1 month, 6 months or 10 months post switch in *ad libitum* (AL) mice and mice switched from 30% dietary restriction (DR) to AL (DR-AL) feeding.** Description: The DR-AL mice had significantly greater food intakes at both 1 month (t = 4.415, *P* = 0.003) and 6 months (t = 5.074, *P* = 0.001) post switch relative to AL controls. No difference in food intake was observed between groups at the final (10-month) timepoint (t = 2.231, *P* = 0.061). The same letter within a timepoint indicated no significant difference between AL and DR-AL mice within the same timepoint. Please note that there were two mice per cage, so histograms show mean (± SEM) daily food intake for two mice within a cage. N = 4 to 5 pairs (that is, 8 to 10 mice) per group. Click here for file
