# Supplementary figures and images for: Evidence of a metabolic memory to early-life dietary restriction in male C57BL/6 mice
Source: Longev Healthspan. 2012 Sep 3;1:2. doi: 10.1186/2046-2395-1-2 (PMC3886256; doi:10.1186/2046-2395-1-2)

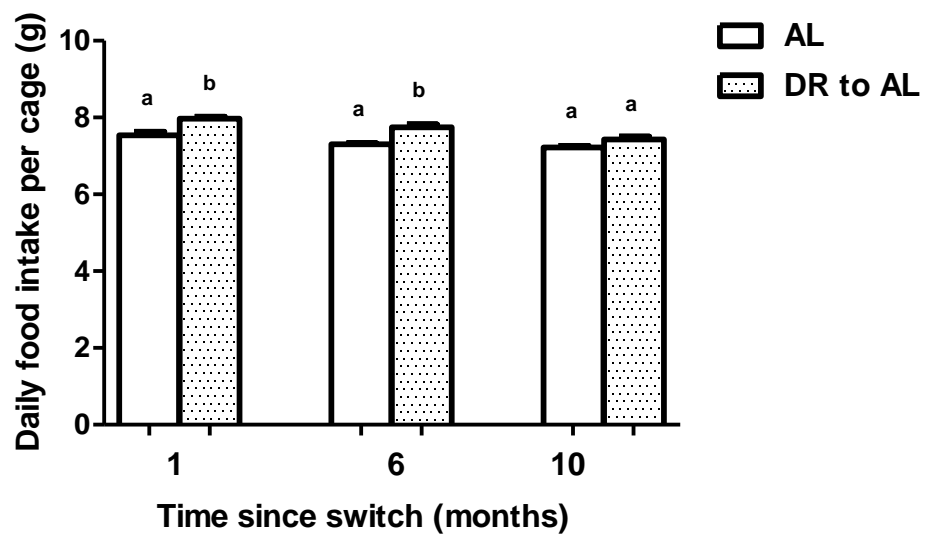

Supplement: Additional file 1: — Title: Mean (± SEM) daily food intake per cage at 1 month, 6 months or 10 months post switch in ad libitum (AL) mice and mice switched from 30% dietary restriction (DR) to AL (DR-AL) feeding. Description: The DR-AL mice had significantly greater food intakes at both 1 month (t = 4.415, P = 0.003) and 6 months (t = 5.074, P = 0.001) post switch relative to AL controls. No difference in food intake was observed between groups at the final (10-month) timepoint (t = 2.231, P = 0.061). The same letter within a timepoint indicated no significant difference between AL and DR-AL mice within the same timepoint. Please note that there were two mice per cage, so histograms show mean (± SEM) daily food intake for two mice within a cage. N = 4 to 5 pairs (that is, 8 to 10 mice) per group. [file 2046-2395-1-2-S1.pdf]
